# Biological solid-state NMR: Integrative across different scientific disciplines

**DOI:** 10.1016/j.yjsbx.2022.100075

**Published:** 2022-09-26

**Authors:** Marc Baldus

**Affiliations:** NMR Spectroscopy, Bijvoet Center for Biomolecular Research, Utrecht University, Padualaan 12, 3584 CH Utrecht, The Netherlands

## Abstract

For almost five decades, solid-state NMR (ssNMR) has been used to study complex biomolecular systems. This article gives a view on how ssNMR methods and applications have evolved during this time period in a broader structural biology context. It also discusses possible directions for additional developments and the future role of ssNMR in a life science context and beyond.

Structural biology traditionally refers to experimental approaches that lead to the determination of a -preferably- three-dimensional structural view of a biomolecule. Rapid advancements in virtually all structural biology methods, i.e., X-ray diffraction (Shi, 2014), NMR, and single-particle Electron microscopy (EM, (Henderson, 2018)) as well as mass spectrometry (MS, (Lössl et al., 2016)) or fluorescence light microscopy (Lerner et al., 2018) have taken place in the last two decades. Computational methods enormously profited from these achievements and, in parallel, advanced to a level from only predicting molecular dynamics and binding events to providing *de-novo* 3D structures of biomolecules and their complexes, particularly in relation to protein-based studies (Jumper et al., 2021). More than 50 years after the establishment of the protein data bank (Bonvin, 2021), these advancements have “”democratized” structural biology for a wider range of research areas and shifted the attention more towards determining how structural information is related to biomolecular function as well as the complex and dynamic molecular space in a native environment.

NMR spectroscopy, and in particular, solid-state NMR spectroscopy (ssNMR), offers a unique tool to address these questions because it can provide structural and dynamical information at the most detailed (i.e., atomic) level. Moreover, ssNMR can be applied in a variety of experimental conditions and it is less sensitive to molecular size limitations as compared to solution-state NMR. These beneficial aspects were first realized by pioneers such as Bob Griffin and Jake Schaefer as well as several others who already demonstrated in the 1970s and 1980s the potential of ssNMR to characterize complex biomolecular systems (Griffin, 1981, Schaefer et al., 1978).

In the last two decades, developments in several areas have greatly expanded the use of ssNMR for such applications. The advent of high and ultra-high field NMR instruments together with high-sensitivity approaches including Dynamic Nuclear Polarization (DNP (Ni et al., 2013)), ^1^H detected ssNMR (Chevelkov et al., 2006, Fricke et al., 2017) and commercial cryoprobes have dramatically improved spectral resolution and sensitivity. In parallel, novel software tools and radio-frequency pulse and data acquisition schemes were designed that support and accelerate various stages of ssNMR-based studies starting with tailoring optimal labelling schemes to spectral assignments and the collection of structural restraints. These procedures have led to a remarkable number of 3D molecular structures determined by ssNMR data alone. Subsequently, such structural studies could be followed by measurements of molecular motion across different time scales and/or the use of ssNMR approaches that allow probing supramolecular structure including solvent exposure, membrane embedding or oligomerization. Many of these advancements have greatly profited from adapting ideas originally developed for biological solution-state NMR in the 1990s. In fact, several of today’s well-known researchers in biological solid-state NMR have started their NMR careers working in solution-state NMR. On the other hand, well established ssNMR concepts such as the use of dipolar couplings have expanded the toolbox of solution NMR studies. Indeed, today’s NMR studies dealing with complex biomolecular systems such as biomolecular complexes, membrane-embedded proteins or molecular condensates profit from integrating NMR data obtained under solution and solid-phase conditions.

Next to the combined use of solid and solution-state NMR, paramagnetic NMR and especially the advent and commercialization of Dynamic Nuclear Polarization instrumentation starting around 2005 strengthened the ties between solid-state NMR and Electron Paramagnetic Resonance (EPR) as well as synthetic and physical chemistry. For example, soluble and tagged paramagnetic probes have become part of the standard toolbox of ssNMR and ab-initio chemical-shift calculations provide a powerful tool in ssNMR studies of small molecules. EPR as well as synthetic chemistry are also of critical relevance to drive the theoretical understanding of different DNP polarization transfer mechanisms and the development of novel DNP agents that optimally perform at increasing magnetic fields and in complex biomolecular settings including cells.

In parallel, progress in biochemistry and chemical biology have triggered the development of novel preparative methods for isotope-labelling of proteins, nucleotides, lipids or carbohydrates. For example, these include cell-free synthesis as well as expression protocols in yeast or human cells that have successfully been used for ssNMR. These advancements have not only expanded the chemical space available to biomolecular ssNMR but also led to the development of innovative reconstitution methods that reduce spectral complexity and enable dissecting atomic details that determine structure and assembly of biomolecular systems. For example, amyloid co-seeding and tailored expression and labelling approaches have opened up novel routes to conduct ssNMR on human cells and tissues (Ghosh et al., 2021, Kaplan et al., 2016). Compared to aforementioned ssNMR studies in the 1970s and 80s, sample preparation methods have also significantly advanced to study entire bacteria as well as algae, fungi, or plant cells (Narasimhan et al., 2021, Ghassemi et al., 2022). These methods enable ssNMR data acquisition in highly complex biomolecular systems at increased spectral sensitivity. In selected cases (Narasimhan et al., 2021), methods were already devised to spectrally zoom in into molecular species that are specifically labeled in an otherwise unlabeled biomolecular background. In addition, spectral filtering techniques and tailored purification methods allow to spectroscopically or chemically dissect molecular entities derived from in-situ preparations.

With the aspects in mind, the molecular complexity that can be tackled by ssNMR has increased considerably. For example, ssNMR studies of microcrystalline or membrane proteins comprising hundreds of amino acids and consisting of several protein subunits have become possible. While structural studies of amyloid proteins have been strong application areas of ssNMR, additional fields have emerged, such as protein assemblies related to bacterial, microtubular and viral complexes. Next to proteins and again similar to solution-state NMR, ssNMR is increasingly used to study nuclei acids and RNA/DNA-protein complexes (Marchanka and Carlomagno, 2019). Given their growing pharmacological relevance and intrinsic dynamic nature, the scope of ssNMR studies on such systems will likely further grow in the future.

At the same time, advancements in ssNMR not only increasingly allow studying biomolecules *in situ* but also provide a powerful means to dissect the intermolecular interactions as well as the influence of the surrounding chemical and dynamical space on biomolecules with great flexibility. For example, bacterial cells largely contain water (70 %) and a diverse chemical space that not only contains proteins (15 %) but also RNA & DNA as well as phospholipids, polysaccharides and small molecules. Again, ssNMR can play a unique role in such studies due to its ability to bridge the gap between classical structural biology approaches and biophysical, biochemical and cell biology methods that provide complementary information. For example, ssNMR can now be applied using in-vitro preparations, cell lysates (under cryogenic conditions), intact cells as well as three-dimensional cell cultures (biofilms, spheroids), offering a powerful means to study the interplay between biomolecular structure and dynamics in relation to the cellular environment and the influence of external stimuli. At the same time, ssNMR provides a powerful link to biophysics and physical chemistry to study molecular folding and recognition events that take place on a wide range of time scales. Indeed, tailored ssNMR approaches have been developed to probe protein disorder and folding, even in the case of large membrane proteins (Xiao et al., 2019) and such methods are also of particular interest for dynamic biomolecular networks including protein hydrogels and condensates. As mentioned above, many of such studies will profit from combining solid and solution NMR methods in synergy with other experimental and in-silico tools (Damman et al., 2019).

Taken together, the aforementioned advancements have allowed to conduct ssNMR studies on systems that were considered intractable in the 1990s. Yet, such “revolutionary” developments also took place in other areas of structural biology, biophysics and cell biology. As a result, the scientific landscape in which solid-state NMR and NMR in general are operating today has changed. Information that previously could only be obtained by NMR or X-ray crystallography may now be accessible via other methods such as EM, MS, single molecule light or atomic force microscopy as well as in-silico modelling. Clearly, each of these methods has intrinsic shortcomings and ssNMR will continue to play an important role in providing high-resolution information in complex systems. At the same time, these broad scientific advancements offer exciting prospects to implement a new generation of ssNMR methods that synergistically use ssNMR data and readouts from complementary methods. For example, in-silico MD trajectories or 3D structural models can be used in the early stages of the ssNMR data acquisition and analysis instead of a full-scale de novo analysis using ssNMR required in the past. Such hybrid approaches will speed up and expand the scope of ssNMR studies. In the next years, we will likely see further advancements on how to design such synergistic approaches in which ssNMR can provide critical information at a faster rate and with higher accuracy. In addition, the introduction of non-uniform sampling methods were early demonstrations of what latest data analysis and machine learning tools may offer to next-generation ssNMR experimentalists. In addition, streamlining the synergistic analysis of ssNMR data and in-situ readouts obtained from high-resolution light microscopy (Kaplan et al., 2016), electron tomography (Baker et al., 2018) or mass spectrometry (Kaplan et al., 2016) will be of considerable interest. Especially in complex native settings, such data will be critical to place NMR results (which will continue to report on molecular ensembles in contrast to single molecule events) in a macromolecular context. For example, the emerging “subcellular protein atlas” based on imaging methods (Kobayashi et al., 2022) (see also https://opencell.czbiohub.org/) will help to interrogate locations of ssNMR target and/or signal enhancement molecules in a cellular context. Next to such “in-situ” information on the nanometer to micrometer scale, tailored purification methods that enable a controlled dissection of cellular entities and maintain cellular organelle substructure will be of interest and may be combined with analytical chemistry methods such as HPLC, GC–MS or immunoblotting to characterize sample preparations in an early stage.

With such a broad portfolio of methods in mind, many future solid-state NMR spectroscopists will likely combine state-of-the-art ssNMR expertise with a multidisciplinary scientific background and/or engage in collaborative team efforts across scientific disciplines. Such contacts will also help to raise awareness of the potential of ssNMR outside the NMR community. For such fields and application areas, “democratizing” ssNMR preparation and data acquisition protocols will be important. Additional advancements in ssNMR sensitivity and resolution will further diminish the required sample amounts (such as the widespread usage of ^19^F NMR) and reduce or completely abandon the need of isotope labelling. The latter aspect will facilitate tracking “native” processes ranging from patient or engineered tissue materials to monitoring bacterial, fungal and plant growth under changing environmental conditions.

With these aspects in mind the potential of ssNMR as a critical player in obtaining an integrative view of complex and functional molecular systems is immense and extends from traditional life science areas such as structural and biophysical chemistry to food and plant science. On the other hand, ssNMR has a strong standing in material science research and “bio-inspired” ssNMR approaches will, yet again, provide a natural link to sustainability research such as in the fields of biotechnology, (bio)materials and biocatalysis.

## Declaration of Competing Interest

The author declares that he has no known competing financial interests or personal relationships that could have appeared to influence the work reported in this paper.

## Data Availability

No data was used for the research described in the article.
